# Supplementary material for: The Acid Ceramidase Is a SARS-CoV-2 Host Factor
Source: Cells. 2022 Aug 15;11(16):2532. doi: 10.3390/cells11162532 (PMC9406565; doi:10.3390/cells11162532)
Supplement: Supplementary file 1 [file cells-11-02532-s001.zip › cells-1832747-supplementary.pdf]

Supporting Information

The Acid Ceramidase is a SARS-CoV-2 Host Factor

Nina Geiger [1], Louise Kersting [2], Jan Schlegel [3,†], Linda Stelz [3], Sofie Fähr [1], Viktoria Diesendorf [1], Valeria Roll [1], Marie Sostmann [1], Eva-Maria König [1], Sebastian Reinhard [3], Daniela Brenner [2], Sibylle Schneider-Schaulies [1], Markus Sauer [3,*], Jürgen Seibel [2,*] and Jochen Bodem [1,*]

[1] Institute of Virology und Immunobiology, Julius-Maximilians-Universität Würzburg, Versbacher Str. 7, 97078 Würzburg, Germany
[2] Institute of Organic Chemistry, Julius-Maximilians-Universität Würzburg, Am Hubland, 97074 Würzburg, Germany
[3] Department of Biotechnology and Biophysics, Biocenter, Julius-Maximilians-Universität Würzburg, Am Hubland, 97074 Würzburg, Germany
* Correspondence: m.sauer@uni-wuerzburg.de (M.S.); seibel@chemie.uni-wuerzburg.de (J.S.); jochen.bodem@vim.uni-wuerzburg.de (J.B.)
† Current Address: Department of Women's and Children's Health, Karolinska Institutet, SciLifeLab, Tomtebodavägen 23B, Solna, 171 65 Stockholm, Sweden.

1. TOXICITY DATA ON HUH7 AND VERO CELLS - INFECTION DATA ON VERO CELLS 2

2. IMAGEJ MACRO FOR DETECTION AND QUANTIFICATION OF FISH-CLUSTERS 2

3. FISH LABELLING OF INFECTED CELLS AFTER 24 H. 3

4. TOXICITY DATA ON MDCK CELLS AND ANALYSES OF LYSOSOMAL PREPARATION OF CELLS INFECTED WITH INFLUENZA VIRUS. 4

5. TOXICITY DATA OF CERANIB-2 AND C6 CERAMIDE ON HUH-7 CELLS 5

6. OLIGONUCLEOTIDE SEQUENCES FOR FISH LABELLING 6

7. SYNTHESIS OF THE FLUOXETINE DERIVATIVES 8

8. NMR SPECTRA OF THE COMPOUNDS 9

9. REFERENCES 13

Toxicity data on Huh7 and Vero cells - infection data on Vero cells

Cellular proliferation assays

The proliferation of cells with and without the compounds was determined by direct automatic cell counting. Cells were seeded on optical plates (CellCarrier-96, PerkinElmer) and counted before the experiments. Then the compounds were added in decreasing concentrations, and the cells were incubated for three days. The cell numbers per well were determined using the PerkinElmer Ensight reader. Only compound concentrations that did not reduce the cell number per well significantly were used for antiviral assays.

A. Toxicity data on Huh-7 cells.

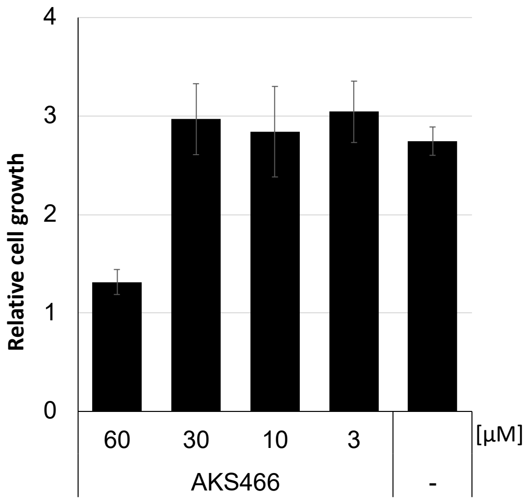

Figure S1. AKS466 shows up to a concentration of 30 µM no toxicity on Huh-7 cells. Huh-7 cells were incubated with the compound AKS466 for 72 hours. After 3 days the relative cell growth was measured.

B. Toxicity data and SARS-CoV-2 infection of Vero cells

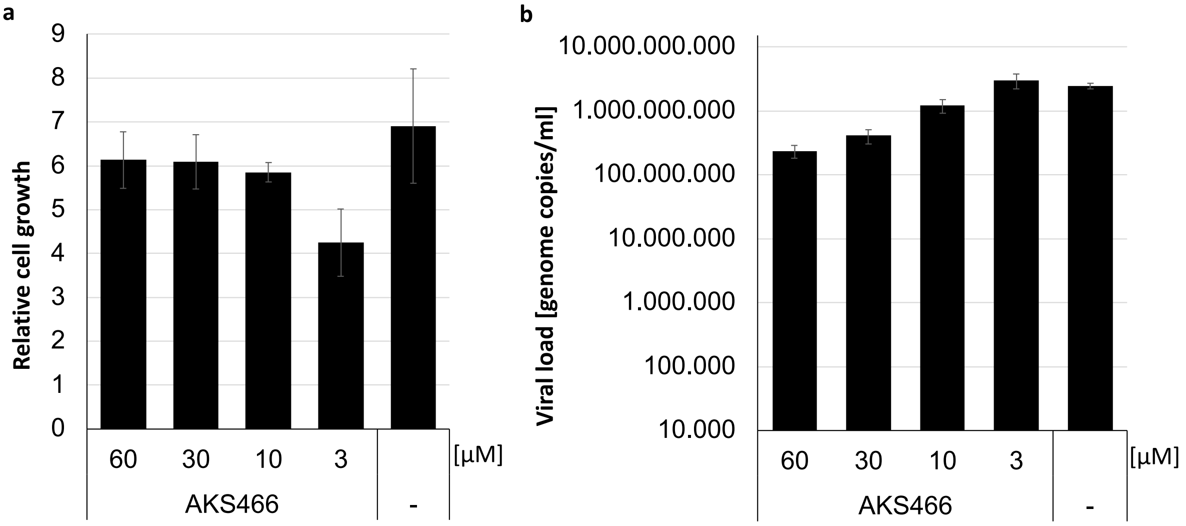

Figure S2. AKS466 inhibits SARS-CoV-2 replication on Vero cells. (**a**) Vero cells were treated with the compound AKS466 for 72 hours, and the cell growth was detected. (**b**) Vero cells were incubated with the compound AKS466 and subsequently infected with 0.5 µl SARS-CoV-2. Cellular supernatants were collected 3 days after infection, and the viral titers were determined by RTqPCR.

ImageJ macro for Detection and quantification of FISH-clusters

```
mypath = File.openDialog("Select a CoronaFISH .czi-file");
run("Bio-Formats", "open=mypath autoscale color_mode=Composite rois_import=[ROI manager] view=Hyperstack stack_order=XYCZT");
rawstack = getTitle();
selectWindow(rawstack);
run("Duplicate...", "duplicate channels=1");
CoronaFISH = getTitle();
selectWindow(rawstack);
close();
selectWindow(CoronaFISH);
run("Z Project...", "projection=[Max Intensity]");
MIPCoronaFISH = getTitle();
run("Duplicate...", " ");
MIPCoronaFISH2 = getTitle();
selectWindow(CoronaFISH);
close();
selectWindow(MIPCoronaFISH);
setAutoThreshold("IsoData dark");
getThreshold(lower, upper);
setOption("BlackBackground", true);
run("Convert to Mask");
run("Watershed");
run("Analyze Particles...", "display exclude clear summarize add in_situ");
selectWindow(MIPCoronaFISH2);
roiManager("multi-measure measure_all");
selectWindow("Results");
saveAs("Results", mypath+"Results_2D-AP-ROIs"+lower+"-"+upper+".csv");
//saveAs("Results", mypath+"Results_"+k+".csv");
roiManager("deselect");
roiManager("Save", mypath+"Results_2D-AP-ROIs.zip");
selectWindow(MIPCoronaFISH);
close();
close("ROI Manager");
close("Results");
close("Summary");
run("Close All");
```

Fish labelling of infected cells after 24 h.

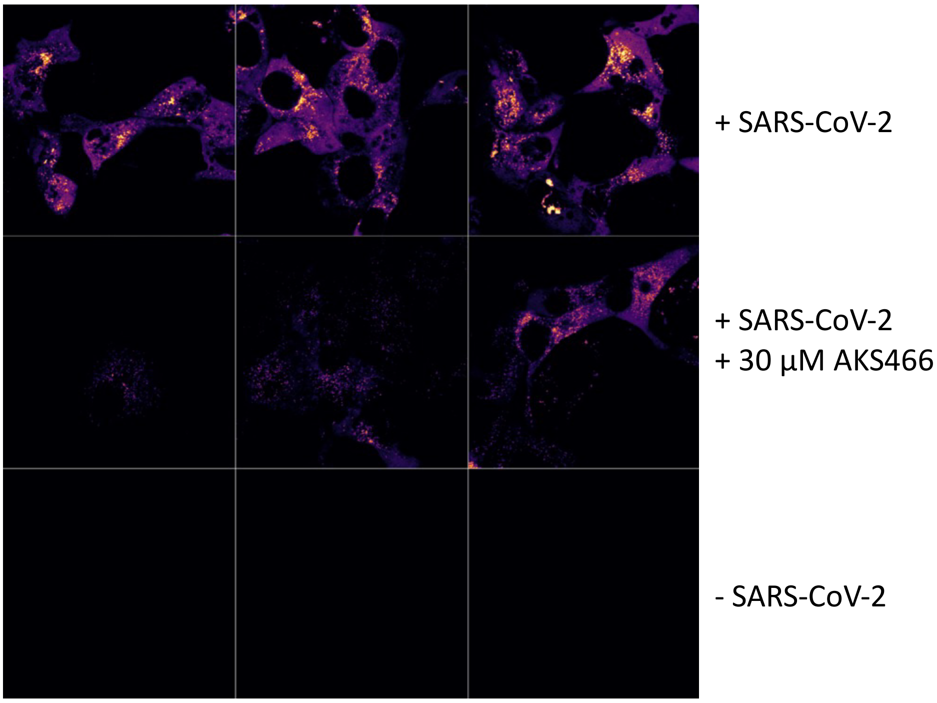

Figure S3. RNA-FISH imaging of infected (SARS-CoV-2) and non-infected (-SARS-CoV-2) Vero-cells after 24 h.

Toxicity data on MDCK cells and analyses of lysosomal preparation of cells infected with influenza virus.

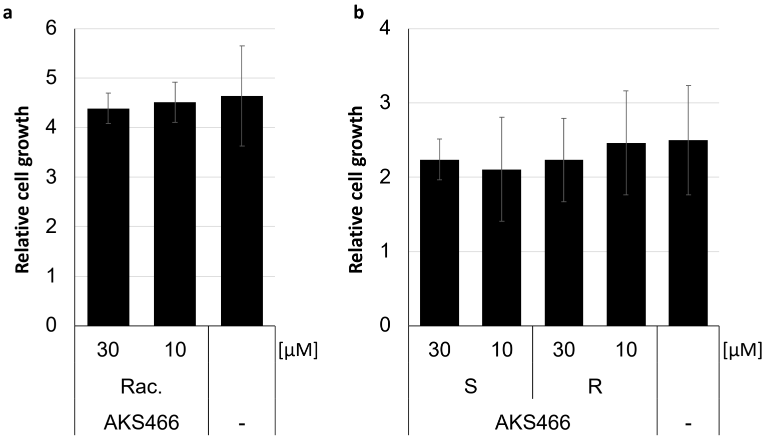

Figure S4. AKS466 shows no cytotoxicity on MDCK cells. (**a**) Racemic AKS466 were incubated on MDCK cells for 3 days, and the cell growth was measured. (**b**) AKS466 as a S- and R-isomere were incubated on MDCK cells for 3 days. Also the relative cell growth was determined.

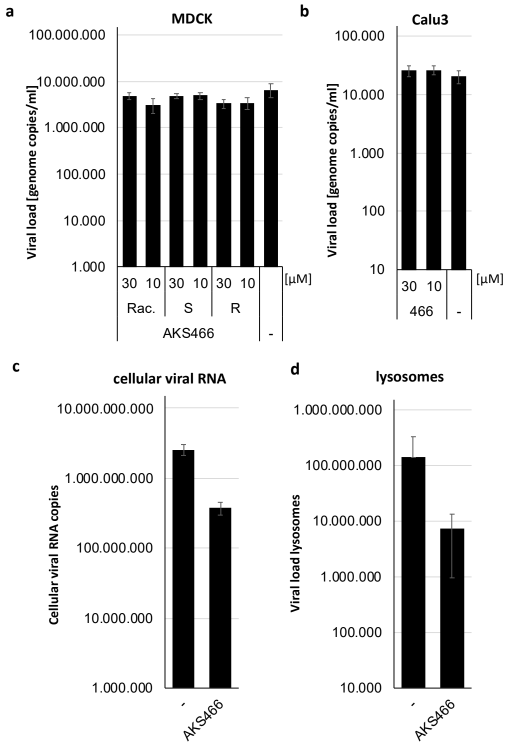

Figure S5. (a) MDCK or **(b)** Calu3 cells were incubated with AKS466 and infected with Influenza virus A. Cell culture supernatants were harvested after 2 days, and viral loads were determined by RTqPCR.**(c)** AKS466 reduces the viral RNA concentration in the cells. Cells were treated and infected with AKS466, cellular RNAs were isolated, and viral transcripts were quantified by RTqPCR. (**c**) MDCK cells were treated with AKS466 and infected with Influenza virus. The cells were lysed, and lysosomes were purified. Viral RNAs were quantified.

Toxicity data of Ceranib-2 and C6 ceramide on Huh-7 cells

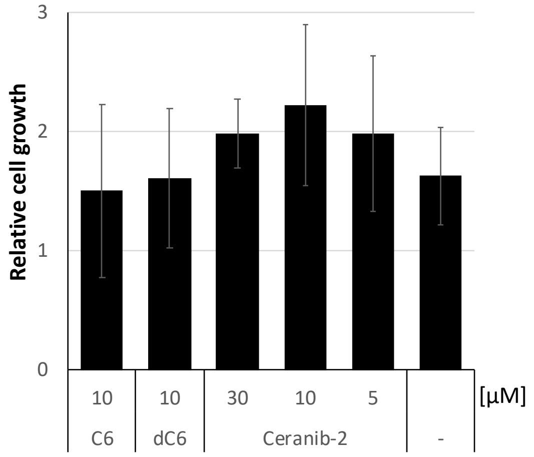

Figure S6. Ceranib-2 and C6 at shows no cytotoxicity on Huh-7 cells. (**a**) C6, dC6 and Ceranib-2 were incubated on Huh-7 cells for 3 days, and the cell growth was measured.

Oligonucleotide sequences for FISH labelling

CGCCTAAGTCAAATGACTTTAGATCGGCGCTTACACTCGGACCTCGTCGACATGCATT
GGTCCACCAAACGTAATGCGGGGTGCATTACACTCGGACCTCGTCGACATGCATT
ATCTTGAAGTTTTCCAAGTGCACTTGCTGTGTTACACTCGGACCTCGTCGACATGCATT
TAACATGACCATGAGGTGCAGTTCGAGCATTTACACTCGGACCTCGTCGACATGCATT
TGTGATCAACCTATCAATTTGCACTTCAGCTTACACTCGGACCTCGTCGACATGCATT
GTTGTTTTGATCGCGCCCCACTGCGTTTACACTCGGACCTCGTCGACATGCATT
TTGAACTGTTGCGACTACGTGATGAGGAACGATTACACTCGGACCTCGTCGACATGCATT
AAGTGTCTCACCACTACGACCGTACTGAATTACACTCGGACCTCGTCGACATGCATT
CGCCTTGTCCTCGAGGGAATTTAAGGTCTTTTACACTCGGACCTCGTCGACATGCATT
AACGCTGAAGCGCTGGGGGCAAATTGTGTTACACTCGGACCTCGTCGACATGCATT
CCGTTAAGCTCACGCATGAGTTCACGGGTTACACTCGGACCTCGTCGACATGCATT
TTTGTTCTGGACCACGTCTGCCGAAAGCTTACACTCGGACCTCGTCGACATGCATT
GTCTTCATAGAACGAACAACGCACTACAAGACTTACACTCGGACCTCGTCGACATGCATT
AAATCTGAAGGAGTAGCATCCTTGATTTCACCTTACACTCGGACCTCGTCGACATGCATT
AGTTCTTTTCTTGTGCAGGGACATAAGTCACATTACACTCGGACCTCGTCGACATGCATT
TAGCACGTGGAACCCAATAGGCACACTTTTACACTCGGACCTCGTCGACATGCATT
GCATTTTGGGGTAAGTAACCACAAGTAGTGGCTTACACTCGGACCTCGTCGACATGCATT
TGAGTTGGTAGTCCCAAAATCTTTGAGGCTACTTACACTCGGACCTCGTCGACATGCATT
GACATTTGTGCGAACAGTATCTACACAAACTCTTACACTCGGACCTCGTCGACATGCATT
ATTGTTTGATGAAGCCAGCATCTGCAAGTGTTTACACTCGGACCTCGTCGACATGCATT
AACGTTCCGTGTACCAAGCAATTTCATGCTTTACACTCGGACCTCGTCGACATGCATT
AGTTGTTATCGACATAGCGAGTGTATGCCCTTACACTCGGACCTCGTCGACATGCATT
GGTCCAACCAGAAGTGATTGTACCCGCTTTACACTCGGACCTCGTCGACATGCATT
TAGGCGTGACAAGTTTCATTATGATCTTGCAGTTACACTCGGACCTCGTCGACATGCATT
GACTGTAAACTACATTCTTGGTGAAATGCAGCTTACACTCGGACCTCGTCGACATGCATT
TGTTCACGGCAGCAGTATACACCCCTTTACACTCGGACCTCGTCGACATGCATT
GCTTGTAAGGTTGCCCTGTTGTCCAGCTTACACTCGGACCTCGTCGACATGCATT
ATTTCCTTTGCACAGGTGACAATTTGTCCACTTACACTCGGACCTCGTCGACATGCATT
GTTTTCAAGCCAGATTCATTATGGTATTCGGCTTACACTCGGACCTCGTCGACATGCATT
CCCTTTGAGTGCGTGACAAATGTTTCACCTTTACACTCGGACCTCGTCGACATGCATT
TCGATGTACTGAATGGGTGATTTAGAACCAGCTTACACTCGGACCTCGTCGACATGCATT
GGAGAGTGCTAGTTGCCATCTCTTTTTGAGGTTACACTCGGACCTCGTCGACATGCATT
TGGGTATGTCACACTCATATGAGTTGTTGACATTACACTCGGACCTCGTCGACATGCATT
TATTGTTATAGCGGCCTTCTGTAAAACACGCTTACACTCGGACCTCGTCGACATGCATT
AAACAGTGCCAAAGATGTTAGTTAGCCACTGCTTACACTCGGACCTCGTCGACATGCATT
CAGCATCAGTAGTGTCAGCAATGTCTCTGTTACACTCGGACCTCGTCGACATGCATT
CAAAGGCAATAGTGCGACCACCCTTACGATTACACTCGGACCTCGTCGACATGCATT
CTTGGTCATAGACACTGGTAGAATTTCTGTGGTTACACTCGGACCTCGTCGACATGCATT
AGTCAACTGAACAACACCACCTGTAATGTAGTTACACTCGGACCTCGTCGACATGCATT
CTGATAACTAGCGCATATACCTGCACCTTACACTCGGACCTCGTCGACATGCATT
ATGTCAAGAATCTCAAGTGTCTGTGGATCACGTTACACTCGGACCTCGTCGACATGCATT
AAAGGCACATTTGGTTGCATTCATTTGGTGACTTACACTCGGACCTCGTCGACATGCATT
CCAATGTACACATAGTGCTTAGCACGTAATCTTACACTCGGACCTCGTCGACATGCATT
CATCCACGCACAATTCAATTAAAGGTGCTGATTACACTCGGACCTCGTCGACATGCATT
ACAGCAACCTGGTTAGAAGTATTTGTTCCTGGTTACACTCGGACCTCGTCGACATGCATT

GTGACAAGCTACAACACGTTGTATGTTTGCGATTACACTCGGACCTCGTCGACATGCATT
AAATTGTGAGTAACAACCAGTGGTGTGTACCCTTACACTCGGACCTCGTCGACATGCATT
TTGTAGCTTGTTTACCACACGTACAAGGTATCTTACACTCGGACCTCGTCGACATGCATT
ACAACAGCAAGTTGCAAACAAAGTGAACACCCTTACACTCGGACCTCGTCGACATGCATT
TACTATGGCAATCAAGCCAGCTATAAAACCTTACACTCGGACCTCGTCGACATGCATT
GAAGTGAATAGGACACGGGTCATCAACTACATTTACACTCGGACCTCGTCGACATGCATT
AGTTCCATCTCTAATTGAGGTTGAACCTCAACTTACACTCGGACCTCGTCGACATGCATT
GGTGCTGACATCATAACAAAAGGTGACTCCTTACACTCGGACCTCGTCGACATGCATT
TATCTAGTAATAGATTACCAGAAGCAGCGTGCTTACACTCGGACCTCGTCGACATGCATT
TAGTTTCTGGATTGAATGACCACATGGAACGCTTACACTCGGACCTCGTCGACATGCATT
ACGTGCCCGCCGAGGAGAATTAGTCTGTTACACTCGGACCTCGTCGACATGCATT
GTTCTTTTCACTGCACTTTGGAAAGTAACACCTTACACTCGGACCTCGTCGACATGCATT
TCCCATTTTTCAGTATAACCACCAATCTGGTTACACTCGGACCTCGTCGACATGCATT
AGTAGGTATAACCACAGCAGTTAAAACACCCTTTACACTCGGACCTCGTCGACATGCATT
TATGCAAAGTATGCCTACTTTTGCTCTGGTTTACACTCGGACCTCGTCGACATGCATT
GTTAACATTTGGGCCGACAACATGAAGACAGTTTACACTCGGACCTCGTCGACATGCATT
CCAGCAATACGAAGATGTCCACGAAGGATCATTACACTCGGACCTCGTCGACATGCATT
AGTAAGCAACTGAATTTTCTGCACCAAGTGACTTACACTCGGACCTCGTCGACATGCATT
ACTGTAATAGTTGTGTCCGTACCAGCTGCTTACACTCGGACCTCGTCGACATGCATT
TATCACCAACATCAGACACTAATGCCTGATTACACTCGGACCTCGTCGACATGCATT
AGTGTAGGCAATGATGGATTGACTAGCTACATTACACTCGGACCTCGTCGACATGCATT
GTCATGTTCAGAAATAGGACTTGTTGTGCCATTTACACTCGGACCTCGTCGACATGCATT
ATGTGATGTTGATATGACATGGTCGTAACAGCTTACACTCGGACCTCGTCGACATGCATT
GTTGAGTTTGAAGGCATCTATGCTATTCTTGGTTACACTCGGACCTCGTCGACATGCATT
CACATGTCTTGGACAGTAAACTACGTCATCAATTACACTCGGACCTCGTCGACATGCATT
GTCCTCTGACTTCAGTACATCAAACGAATTTGTTACACTCGGACCTCGTCGACATGCATT
TGAACAACATCACCCACTATATATGGAGCATTACACTCGGACCTCGTCGACATGCATT
CTTATTTAAGGCTCCTGCAACACCTCCTTACACTCGGACCTCGTCGACATGCATT
CACAGGCGAACTCATTTACTTCTGTACCGATTACACTCGGACCTCGTCGACATGCATT
AACGGTCAAAGAGTTTTAACCTCTCTTCCGTGTTACACTCGGACCTCGTCGACATGCATT
GTGTTGAACCTTTCTACAAGCCGCATTAATCTTACACTCGGACCTCGTCGACATGCATT
ACCAAGGTCACGGGGTGTCATGTTTTCAACTTACACTCGGACCTCGTCGACATGCATT
CGCAGGCAAGATTATCCATTCCCTGCTTACACTCGGACCTCGTCGACATGCATT
ATTTGACAGCAGAATTGGCCCTTAAAGCTGTTTACACTCGGACCTCGTCGACATGCATT
TCCAGTTGGTAATTCCATATGGTGCATGTAACTTACACTCGGACCTCGTCGACATGCATT
GGCAGTACAGACAACACGATGCACCACTTACACTCGGACCTCGTCGACATGCATT
CCCACTTTAAGTGGTCCATTAGTAGCTATGTATTACACTCGGACCTCGTCGACATGCATT
ACAAAGCACTCGTGGACAGCTAGACACCTTACACTCGGACCTCGTCGACATGCATT
AACGTGTGTCAGGGCGTAAACTTTCATAAGCATTACACTCGGACCTCGTCGACATGCATT
AACTGTTATTGCCTGACCAGTACCAGTGTTTACACTCGGACCTCGTCGACATGCATT
GTTGTACCACAAGTTACTTGTACCATACAACCTTACACTCGGACCTCGTCGACATGCATT
GCTTTAGGGTTACCAATGTCGTGAAGAACTGGTTACACTCGGACCTCGTCGACATGCATT
ACACTCAAGAACGTCTTTCTGTATGGTAGGATTTACACTCGGACCTCGTCGACATGCATT
GAAGCCAATCCATGCACGTACATGTCTTATTACACTCGGACCTCGTCGACATGCATT
AGGAATGAACCCTTAATAGTGAAATTGGGCCTTTACACTCGGACCTCGTCGACATGCATT
TGTCATTAGTATAACTACCACCACGCTGGTTACACTCGGACCTCGTCGACATGCATT

AAGTCACCATTAGTTGTGCGTAATATCGTGCCTTACACTCGGACCTCGTCGACATGCATT
AAGATGGATGGAACCATTCTTCACTGTAACACTTACACTCGGACCTCGTCGACATGCATT
TAAAGCTCACAAGTAGCGAGTGTTATCAGTGCTTACACTCGGACCTCGTCGACATGCATT
ACCTCCAAGAGCTAGCTTTTGTTGTATAAACCTTACACTCGGACCTCGTCGACATGCATT
CCTCTACAGTGTAACCATTTAAACCCTGACTTACACTCGGACCTCGTCGACATGCATT

Synthesis of the Fluoxetine derivatives

The synthesis of racemic **AKS466** and the two enantiomers followed the same protocol starting from racemic or enantiomerically pure (S)- or (R)-3-chloro-1-phenylpropan-1-ol. The enantiomeric starting materials were purchased from TCI and had an enantiomeric excess of 98% or higher.

2-(3-Hydroxy-3-phenylpropyl)isoindoline-1,3-dione

3-Chloro-1-phenylpropan-1-ol (949 mg, 5.56 mmol, 1.0 equiv) and potassium phthalimide (1.24 g, 6.67 mmol, 1.2 equiv) were dissolved in anhydrous DMF (15 mL) and stirred for 23 h at 90 °C. H_2O (100 mL) was added, and the mixture was extracted with EtOAc (3 × 100 mL). The combined organic layers were washed with brine (2 × 150 mL) and dried over $MgSO_4$. The solvent was removed under reduced pressure, and the crude product was purified by column chromatography (CH/EtOAc = 2/1) to afford the title compound (1.49 g, 5.30 mmol, 95%) as a colorless solid.

***R*f:** 0.36 (pentane/Et_2O = 2/1); ^{1}**H NMR** (400 MHz, $CDCl_3$): δ 7.87 – 7.81 (m, 2H), 7.75 – 7.69 (m, 2H), 7.37 – 7.27 (m, 4H), 7.24 – 7.18 (m, 1H), 4.68 (dd, ^3J = 7.9 Hz, 5.4 Hz, 1H), 3.95 – 3.86 (m, 2H), 2.81 (br s, 1H, O*H*), 2.16 – 2.01 (m, 2H) ppm.; ^{13}**C NMR** (100 MHz, $CDCl_3$): δ 168.9, 143.6, 134.1, 132.1, 128.5, 127.6, 125.7, 123.4, 71.3, 37.7, 35.0 ppm; **MS** (ESI+): *m/z* calcd for $C_{17}H_{15}NO_3$ $[M+Na]^+$ 304.0944, found 304.0933, $|\Delta m/z|$ = 3.7 ppm.

2-(3-Phenyl-3-(4-(trifluoromethyl)phenoxy)propyl)isoindoline-1,3-dione

Triphenylphosphine (1.12 g, 4.27 mmol, 1.2 equiv) was dissolved in anhydrous THF (15 mL). Diisopropyl azodicarboxylate (838 µL, 863 mg, 4.27 mmol, 1.2 equiv) was added, and the solution was stirred for 20 min. 4-(Trifluoromethyl)phenol (577 mg, 3.56 mmol, 1.0 equiv) was added, and the solution was stirred for 5 h. 2-(3-Hydroxy-3-phenylpropyl)isoindoline-1,3-dione (1.00 g, 3.56 mmol, 1.0 equiv) was added, and the mixture was stirred for 5 d. The solvent was removed under reduced pressure, and the crude product was purified by column chromatography (CH/EtOAC = 10/1, then CH/DCM = 1/2) to afford the title compound (1.07 g, 2.52 mmol, 71%) as a white solid.

***R*f:** 0.24 (CH/EE = 10/1); ^{1}**H NMR** (400 MHz, $CDCl_3$): δ 7.84 – 7.78 (m, 2H), 7.73 – 7.68 (m, 2H), 7.40 – 7.36 (m, 2H), 7.36 – 7.28 (m, 4H), 7.26 – 7.20 (m, 1H), 6.83 – 6.77 (m, 2H), 5.27 (dd, ^3J = 8.8 Hz, 3.9 Hz, 1H), 4.03 – 3.85 (m, 2H), 2.49 -2.37 (m, 1H), 2.27 – 2.15 (m, 1H) ppm; ^{13}**C NMR** (100 MHz, $CDCl_3$): δ 168.4, 160.2, 140.3, 134.0, 132.2, 128.9, 128.1, 126.8 (q, $^4J_{C\text{-}F}$ = 3.8 Hz), 125.8, 124.5 ($^1J_{C\text{-}F}$ = 270.6 Hz), 123.4, 122.9 (q, $^2J_{C\text{-}F}$ = 32.5 Hz), 115.7, 78.5, 37.2, 35.2 ppm; **MS** (ESI+): *m/z* calcd for $C_{24}H_{18}F_3NNaO_3$ $[M+Na]^+$ 448.1131, found 448.1132, , $|\Delta m/z|$ = 0.2 ppm.

The spectroscopic data are in agreement with those reported in the literature.[1]

3-Phenyl-3-(4-(trifluoromethyl)phenoxy)propan-1-amine (Norfluoxetine)

2-(3-Phenyl-3-(4-(trifluoromethyl)phenoxy)propyl)isoindoline-1,3-dione (200 mg, 470 µmol, 1.0 equiv) was dissolved in DCM (1.5 mL) and MeOH (1.5 mL) and hydrazine monohydrate (57.7 µL, 58.8 mg, 1.18 mmol, 2.5 equiv) was added. The solution was stirred for 2 d at rt. H_2O (30 mL) was added, and the aqueous phase was extracted with DCM (3 × 30 mL). NaOH solution was added to the aqueous phase until the pH reached ~ 11 and extracted with DCM (2 × 50 mL) again. The combined organic layers were washed with

brine (100 mL) and dried over $MgSO_4$. The solvent was removed under reduced pressure to afford Norfluoxetine (138 mg, AKS466 µmol, 99%) as a colourless oil. The crude product was used in the following step without further purification. The product can be quantitatively transformed in its HCl salt by stirring in DCM and ethanolic HCl solution.

^{1}H NMR (400 MHz, $CDCl_3$): δ 7.45–7.40 (m, 2H), 7.38–7.30 (m, 4H, *H*-2), 7.30–7.24 (m, 1H), 6.94 – 6.87 (m, 2H), 5.31 (dd, ^3J = 8.4 Hz, ^3J = 4.6 Hz, 1H), 2.89 (dd, ^3J = 7.2 Hz, ^3J = 6.5 Hz, 2H), 2.22–2.11 (m, 1H), 2.02–1.91 (m, 1H) ppm; **^{13}C NMR** (100 MHz, $CDCl_3$): δ 160.5, 141.0 , 128.9, 128.0, 126.8 (q, $^3J_{C\text{-}F}$ = 3.7 Hz), 125.8, 124.4 ($^1J_{C\text{-}F}$ = 271.8 Hz), 122.9 ($^2J_{C\text{-}F}$ = 32.7 Hz), 115.8, 78.4, 41.9, 38.5 ppm; **MS** (ESI+): *m/z* calcd for $C_{16}H_{17}F_3NO$ $[M+H]^+$ 296.1257, found 296.1255, $|\Delta m/z|$ = 0.7 ppm.

The spectroscopic data are in agreement with those reported in the literature.[1]

6-Azido-N-(3-phenyl-3-(4-(trifluoromethyl)phenoxy)propyl)hexanamide (AKS466)

6-Azidohexanoic acid (297 mg, 1.89 mmol, 1.0 equiv) and HATU (862 mg, 2.27 mmol, 1.2 equiv) were dissolved in anhydrous DMF (10 mL). The solution was cooled to 0 °C, and anhydrous triethylamine (527 µL, 382 mg, 3.78 mmol, 2.0 equiv) was added. The solution was stirred for 10 min at this temperature. Then, Norfluoxetine (627 mg, 1.89 mmol, 1.0 equiv) was added, the solution was allowed to warm to rt and stirred for 21 h. Sat. aq. NH_4Cl solution (20 mL) was added, and the aqueous phase was extracted with EtOAc (3 × 50 mL) and DCM (2 × 50 mL). The organic layers were washed with brine (2 × 150 mL), dried over $MgSO_4$, and the solvent was removed under reduced pressure. The crude product was purified by column chromatography (CH/EtOAc = 1/1) to afford **AKS466** (701 mg, 1.61 mmol, 85%) as a colourless oil.

***R_f*:** 0.28 (CH/EtOAc = 1/1); **^{1}H NMR** (400 MHz, $CDCl_3$): δ 7.47–7.41 (m, 2H, *H*-3′), 7.38–7.25 (m, 5H, *H*-1–3), 6.91–6.85 (m, 2H, *H*-2′), 5.59 (br s, 1H, N*H*), 5.24 (dd, ^3J = 7.8 Hz, ^3J = 4.7 Hz, 1H, *H*-5), 3.46 (td, ^3J = 6.2 Hz, ^3J = 5.8 Hz, 2H, *H*-7), 3.26 (t, ^3J = 6.8 Hz, 2H, *H*-13), 2.24–2.09 (m, 2H, *H*-6), 2.14 (t, ^{3}J = 7.5 Hz, 2H, *H*-9, superimposed with *H*-6 signal), 1.68–1.54 (m, 4H, *H*-10, *H*-12), 1.43–1.33 (m, 2H, *H*-11) ppm; **^{13}C NMR** (100 MHz, $CDCl_3$): δ 172.9 (*C*-8), 160.2 (*C*-1′), 140.3 (*C*-4), 129.0 (2C, *C*-2/*C*-3), 128.2 (*C*-1), 126.9 (q, $^3J_{C\text{-}F}$ = 3.7 Hz, 2C, *C*-3′), 125.7 (2C, *C*-2/*C*-3), 124.3 (q, $^1J_{C\text{-}F}$ = 271.2 Hz, *C*-5′), 123.2 (q, $^2J_{C\text{-}F}$ = 32.7 Hz, *C*-4′), 115.8 (2C, *C*-2′), 78.9 (*C*-5), 51.3 (*C*-13), 38.2 (*C*-6), 36.7 (*C*-7), 36.4 (*C*-9), 28.7 (*C*-10/*C*-12), 26.4 (*C*-11), 25.2 (*C*-10/*C*-12) ppm; **MS** (ESI+): *m/z* calcd for $C_{22}H_{25}F_3N_4NaO_2$ [M+Na]+ 457.1822, found 457.1816, $|\Delta m/z|$ = 1.3 ppm.

NMR spectra of the compounds

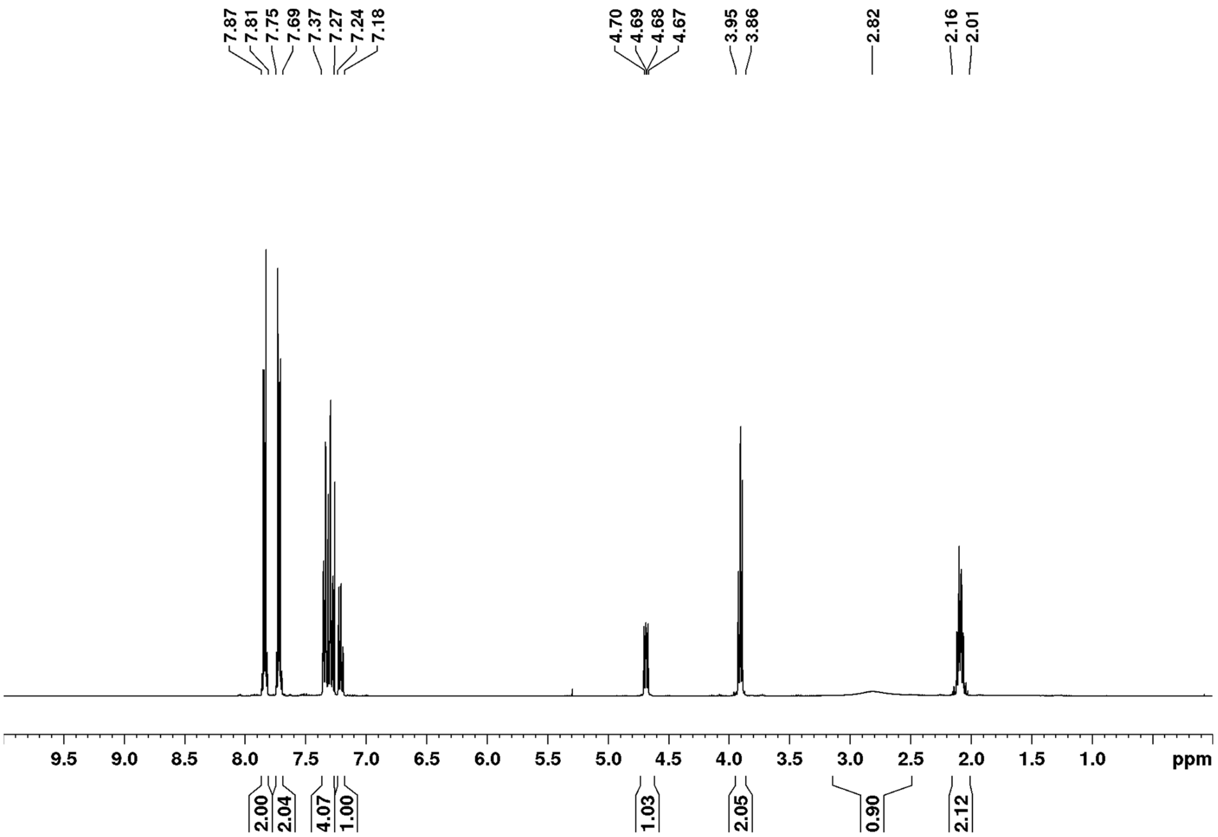

^{1}H NMR spectrum (400 MHz, $CDCl_3$) of 2-(3-Hydroxy-3-phenylpropyl)isoindoline-1,3-dione.

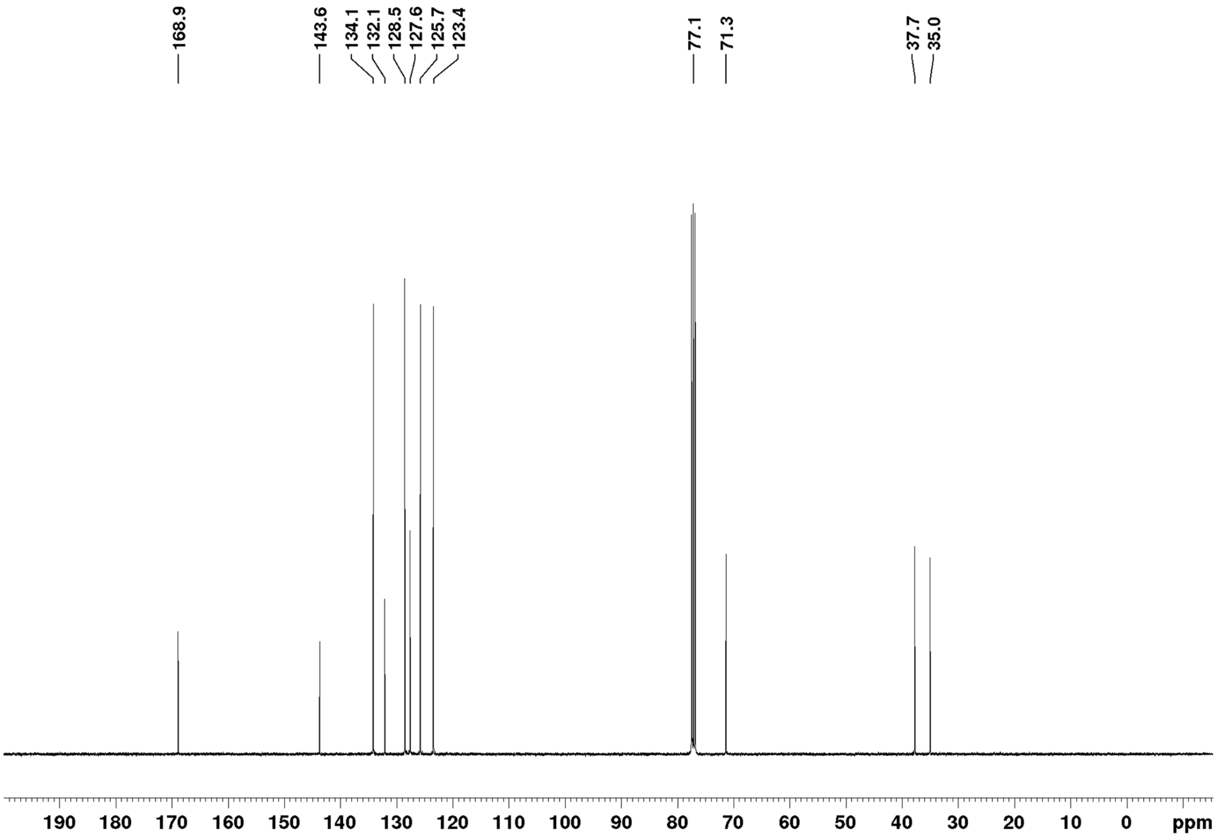

^{13}C NMR spectrum (100 MHz, $CDCl_3$) of 2-(3-Hydroxy-3-phenylpropyl)isoindoline-1,3-dione.

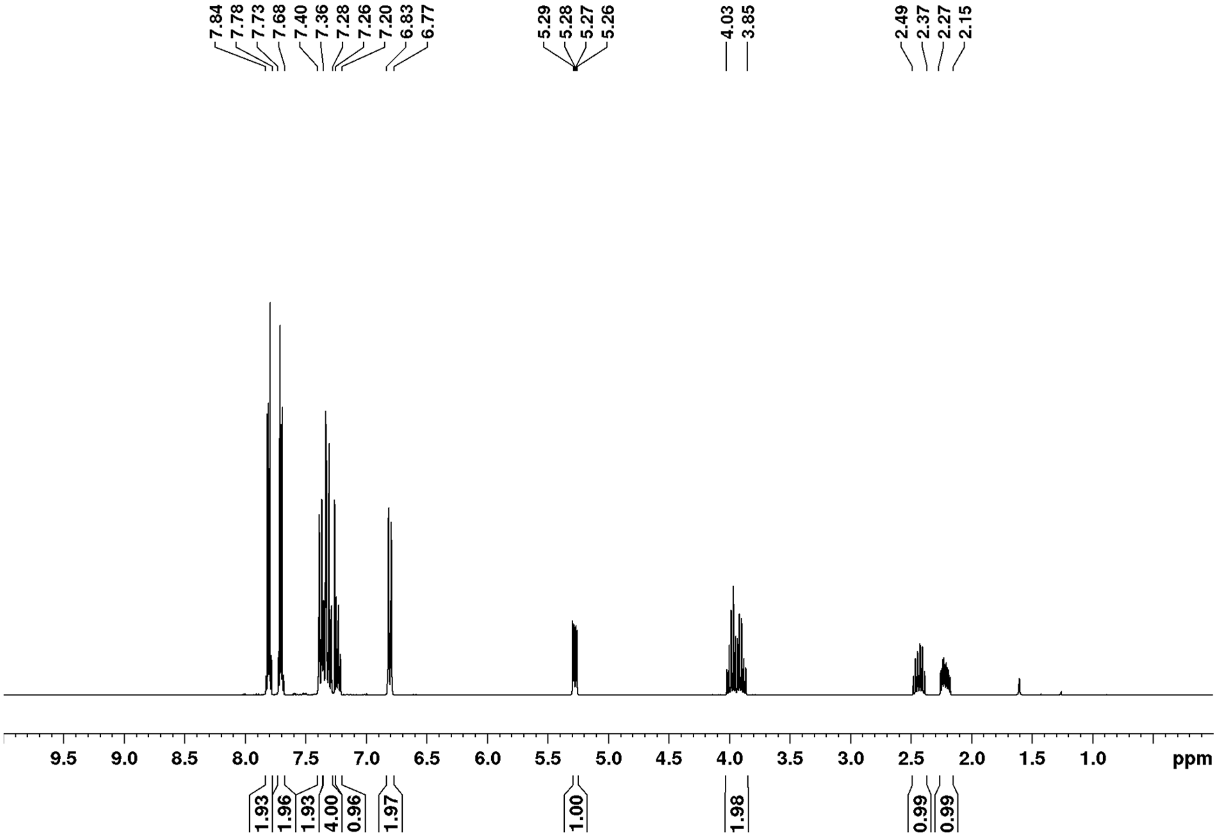

^{1}H NMR spectrum (400 MHz, $CDCl_3$) of 2-(3-Phenyl-3-(4-(trifluoromethyl)phenoxy)propyl)-isoindoline-1,3-dione.

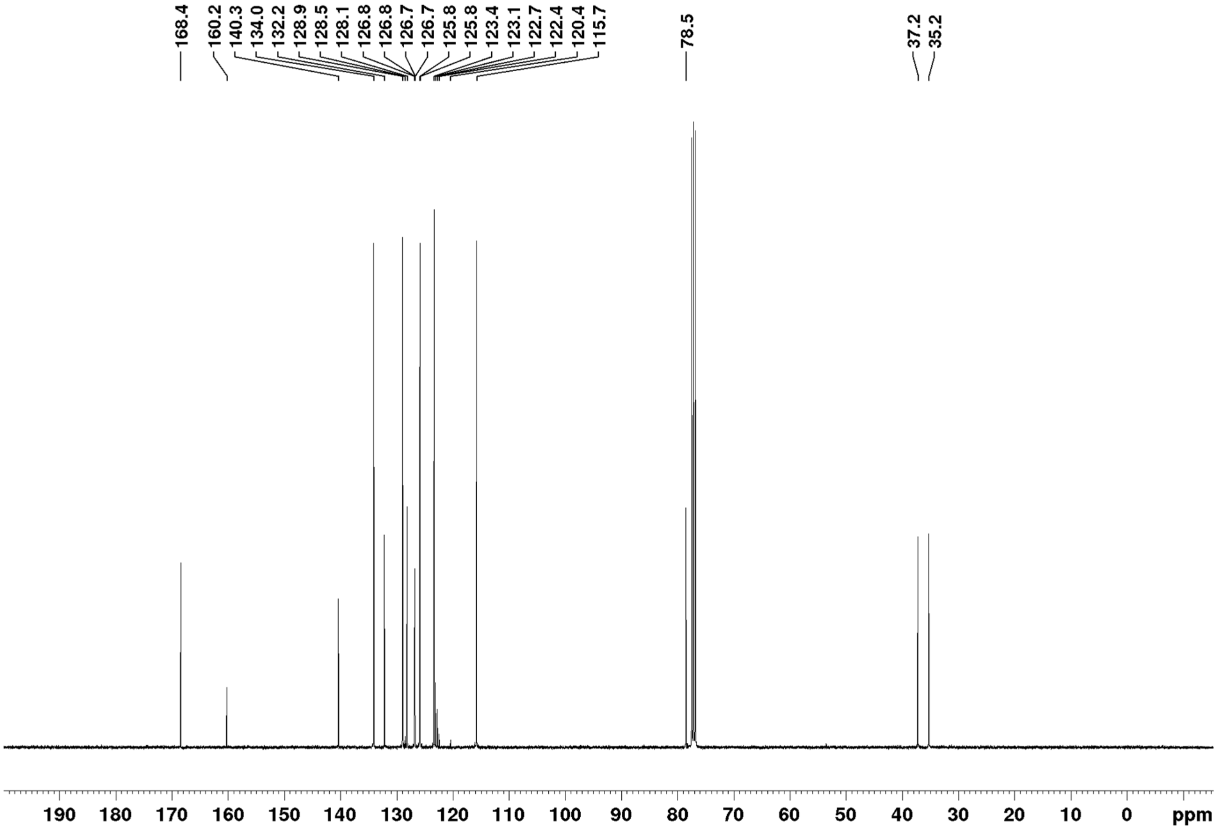

^{13}C NMR spectrum (100 MHz, $CDCl_3$) of 2-(3-Phenyl-3-(4-(trifluoromethyl)phenoxy)propyl)-isoindoline-1,3-dione.

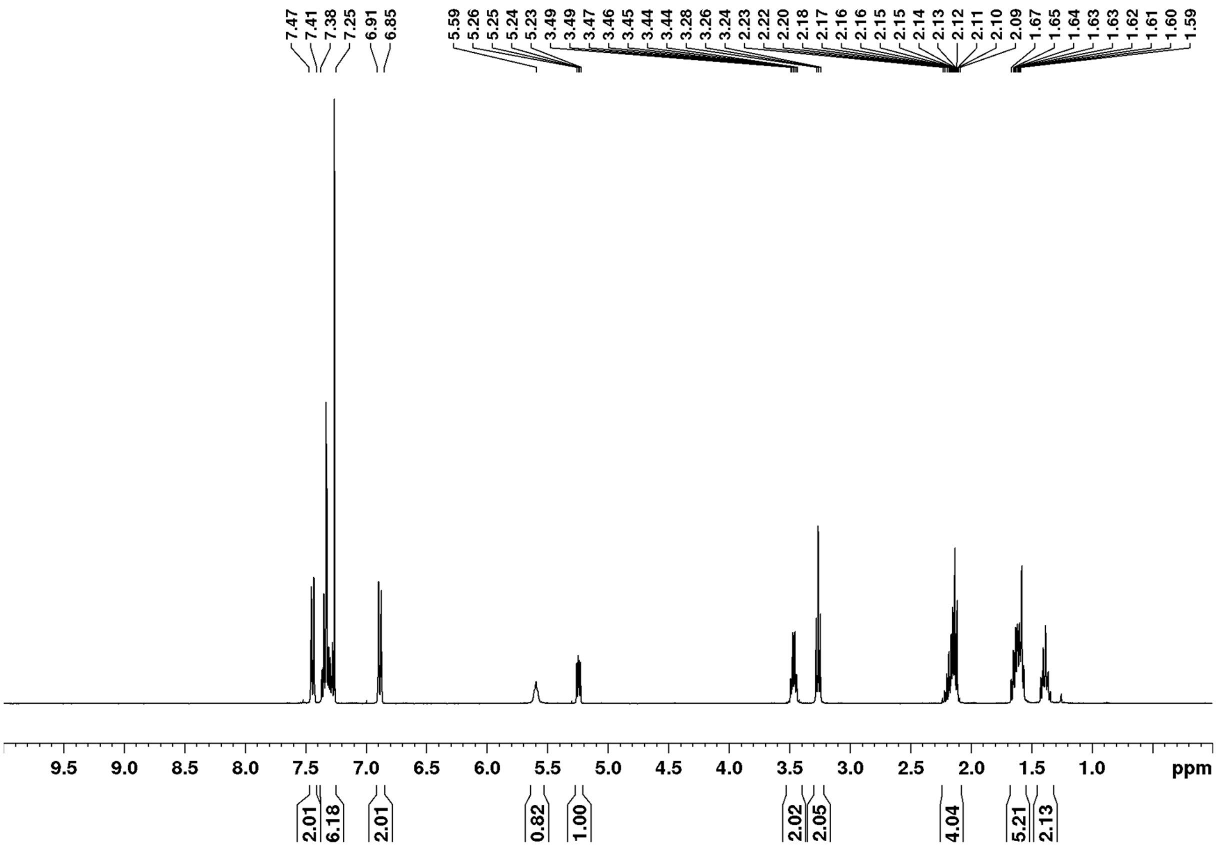

^{1}H NMR spectrum (400 MHz, CDCl3) of compound AKS466.

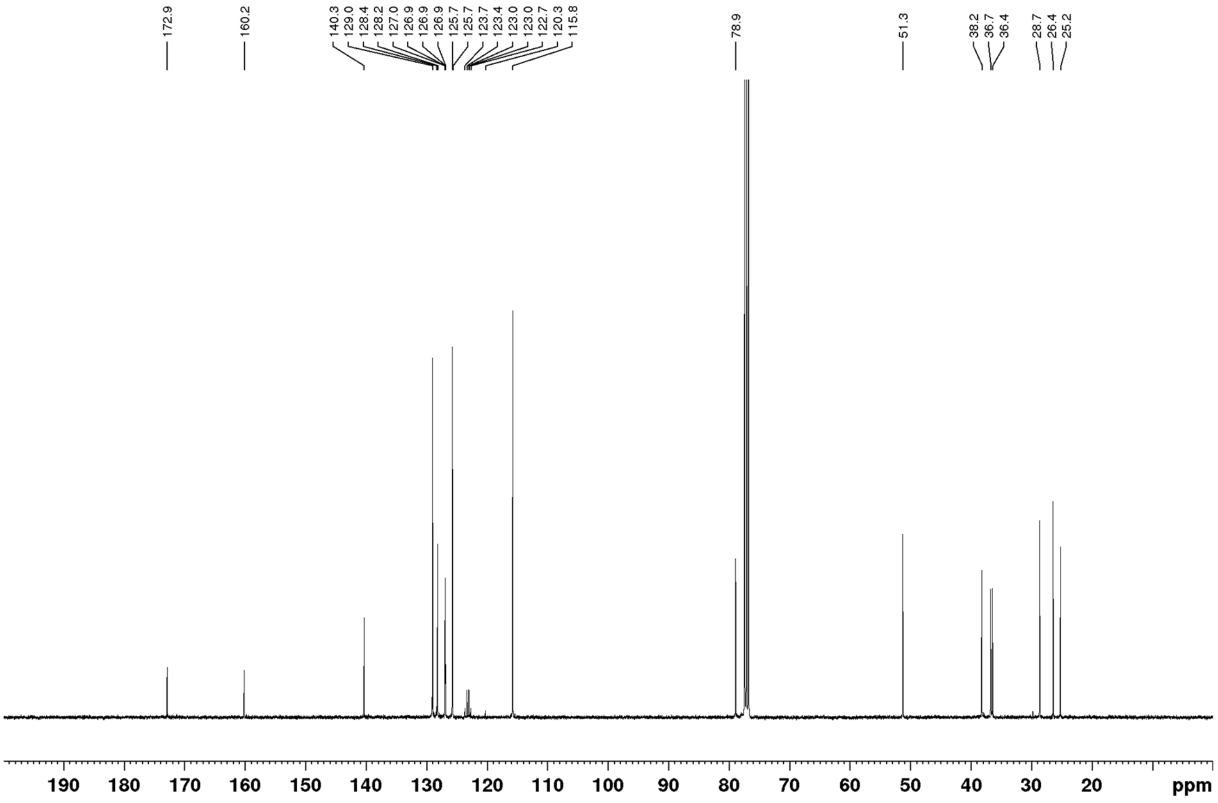

[13.] C NMR spectrum (100 MHz, $CDCl_3$) of compound AKS466.

References.

1 Cashman, J. R., Voelker, T., Johnson, R. & Janowsky, A. Stereoselective inhibition of serotonin re-uptake and phosphodiesterase by dual inhibitors as potential agents for depression. *Bioorg Med Chem* **17**, 337-343, doi:10.1016/j.bmc.2008.10.065 (2009).
